# Cryptic Genetic Diversity Is Paramount in Small-Bodied Amphibians of the Genus *Euparkerella* (Anura: Craugastoridae) Endemic to the Brazilian Atlantic Forest

**DOI:** 10.1371/journal.pone.0079504

**Published:** 2013-11-01

**Authors:** Luciana A. Fusinatto, João Alexandrino, Célio F. B. Haddad, Tuliana O. Brunes, Carlos F. D. Rocha, Fernando Sequeira

**Affiliations:** 1 Departamento de Ecologia, Instituto de Biologia Roberto Alcantara Gomes, Universidade do Estado do Rio de Janeiro, Rio de Janeiro, Rio de Janeiro, Brasil; 2 Departamento de Ciências Biológicas, UNIFESP - Universidade Federal de São Paulo, Campus Diadema, Diadema, São Paulo, Brasil; 3 Departamento de Zoologia, Instituto de Biociências, Universidade Estadual Paulista, Rio Claro, São Paulo, Brasil; 4 CIBIO/UP, Centro de Investigação em Biodiversidade e Recursos Genéticos da Universidade do Porto, Vairão, Portugal; 5 Departamento de Biologia, Faculdade de Ciências da Universidade do Porto, Porto, Portugal; Consiglio Nazionale delle Ricerche (CNR), Italy

## Abstract

Morphological similarity associated to restricted distributions and low dispersal abilities make the direct developing “*Terrarana*” frogs of the genus *Euparkerella* a good model for examining diversification processes. We here infer phylogenetic relationships within the genus *Euparkerella*, using DNA sequence data from one mitochondrial and four nuclear genes coupled with traditional Bayesian phylogenetic reconstruction approaches and more recent coalescent methods of species tree inference. We also used Bayesian clustering analysis and a recent Bayesian coalescent-based approach specifically to infer species delimitation. The analysis of 39 individuals from the four known *Euparkerella* species uncovered high levels of genetic diversity, especially within the two previously morphologically-defined *E. cochranae* and *E. brasiliensis*. Within these species, the gene trees at five independent loci and trees from combined data (concatenated dataset and the species tree) uncovered six deeply diverged and geographically coherent evolutionary units, which may have diverged between the Miocene and the Pleistocene. These six units were also uncovered in the Bayesian clustering analysis, and supported by the Bayesian coalescent-based species delimitation (BPP), and Genealogical Sorting Index (GSI), providing thus strong evidence for underestimation of the current levels of diversity within *Euparkerella*. The cryptic diversity now uncovered opens new opportunities to examine the origins and maintenance of microendemism in the context of spatial heterogeneity and/or human induced fragmentation of the highly threatened Brazilian Atlantic forest hotspot.

## Introduction

The Neotropics harbor one of the highest levels of biodiversity on earth [1], but only a fraction of this biodiversity has been described [2]. Amphibians are among the vertebrates in which species discovery has increased considerably in the last decades [3–7]. The search for new species in poorly known regions, the use of molecular data and of integrative approaches (combining natural history with genetic, morphological, and ecological data) all have contributed to increasing knowledge on amphibian biodiversity [8–12]. Species discovery has often been accompanied by challenging systematics and problematic taxonomies for several groups of amphibians. Morphological conservatism often associated to highly homoplastic traits is commonly mentioned as the cause for discordance between phylogenetic relationships inferred from molecular and morphological data [13–15]. The case of the Neotropical direct developing anurans of the taxon *Terrarana* is paradigmatic of this taxonomic complexity [5]. The taxonomy of this clade, composed by almost a thousand species distributed in four families (Ceuthomantidae, Brachycephalidae, Eleutherodactylidae and Craugastoridae) [16], has been controversial due to scarcity of diagnostic traits and high levels of trait plasticity and convergence [5,16–22]. Recent phylogenetic reconstructions with multimarker DNA data examined the general taxonomy of *Terrarana* [5,16,19], but the evolutionary history and phylogenetic relationships of several clades within this taxon remain largely unknown. 

**Figure 1:**
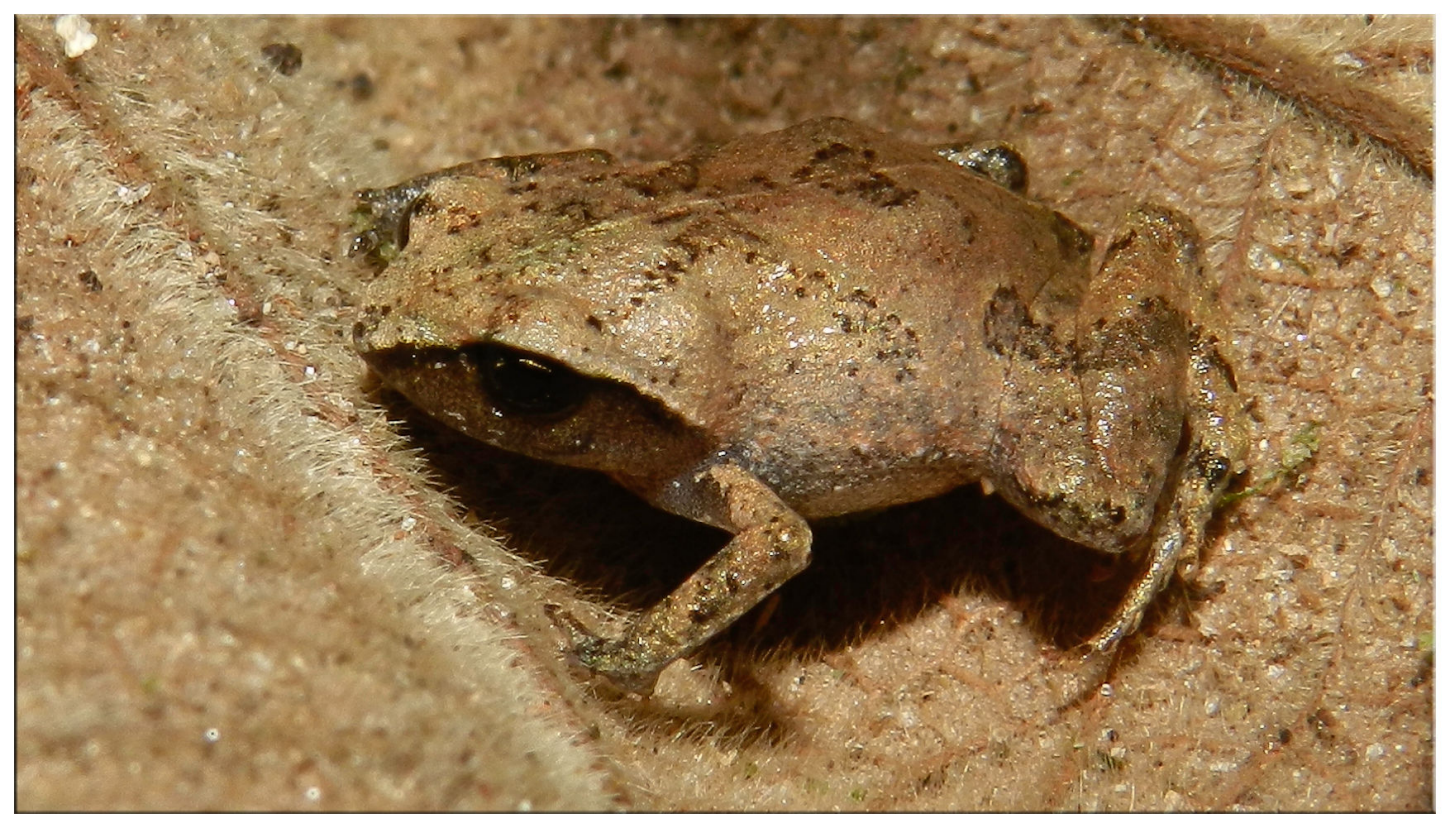
*Euparkerella brasiliensis*. From Cachoeiras de Macacu, Rio de Janeiro State. Locality 5d. (Photo: L. A. Fusinatto) .

The genus *Euparkerella* (Craugastoridae) is one of the least known of the *Terrarana* [16,19]. It currently consists of four morphologically-based species - *E. brasiliensis* (Parker, 1926) (Figure 1), *E. cochranae* Izecksohn, 1988, *E. robusta* Izecksohn, 1988, and *E. tridactyla* Izecksohn, 1988. These species, distinguishable only by cryptic morphological traits [23], are restricted to small areas of Atlantic forest in southeastern Brazil [23,24]. One of the most impressive characteristic of *Euparkerella* species is their small body size (maximum SVL around 22 mm) with extremely reduced digits compared to those of their larger relatives [19], features that are commonly associated with the process of miniaturization [26]. The reduction of adult body size is a recurrent tendency in the evolution of amphibians, being strongly associated with terrestrial life history [25,26]. Small body size and associated traits (e.g., digit reduction) were recently suggested to be innovative morphological features resulting from microhabitat adaptation, that would reduce the vagility and the physiological tolerance of species, ultimately concurring to geographic range restriction and fragmentation, and higher population structure and diversification rates [26,27]. So, morphological similarity and restricted distributions with predicted low dispersal abilities make the genus *Euparkerella* a good model for examining diversification processes. 

In the last decades several new genera and species of amphibians were recognized and described for the Neotropics as a result of increased sampling effort and taxonomic reevaluations using molecular data [5,28–30], but knowledge remains very poor on within genus species genetic diversification and evolutionary history. We here contribute to reduce this knowledge gap by examining patterns and levels of genetic diversity and inferring the phylogenetic relationships within the genus *Euparkerella*. To this purpose, we use DNA sequence data from both mitochondrial and nuclear genes coupled with traditional Bayesian phylogenetic reconstruction methods and more recent coalescent approaches of species tree inference. In addition, we investigate species-level biological diversity applying several methods [31] to delimit putative novel evolutionary units within the most extensively sampled *Euparkerella* species (*E. brasiliensis*, *E. cochranae*) using Bayesian cluster assignment analyses, the Genealogical Sorting Index, and in particular a recent Bayesian coalescent species delimitation approach. 

## Materials and Methods

### Ethics Statement and Sampling

**Figure 2:**
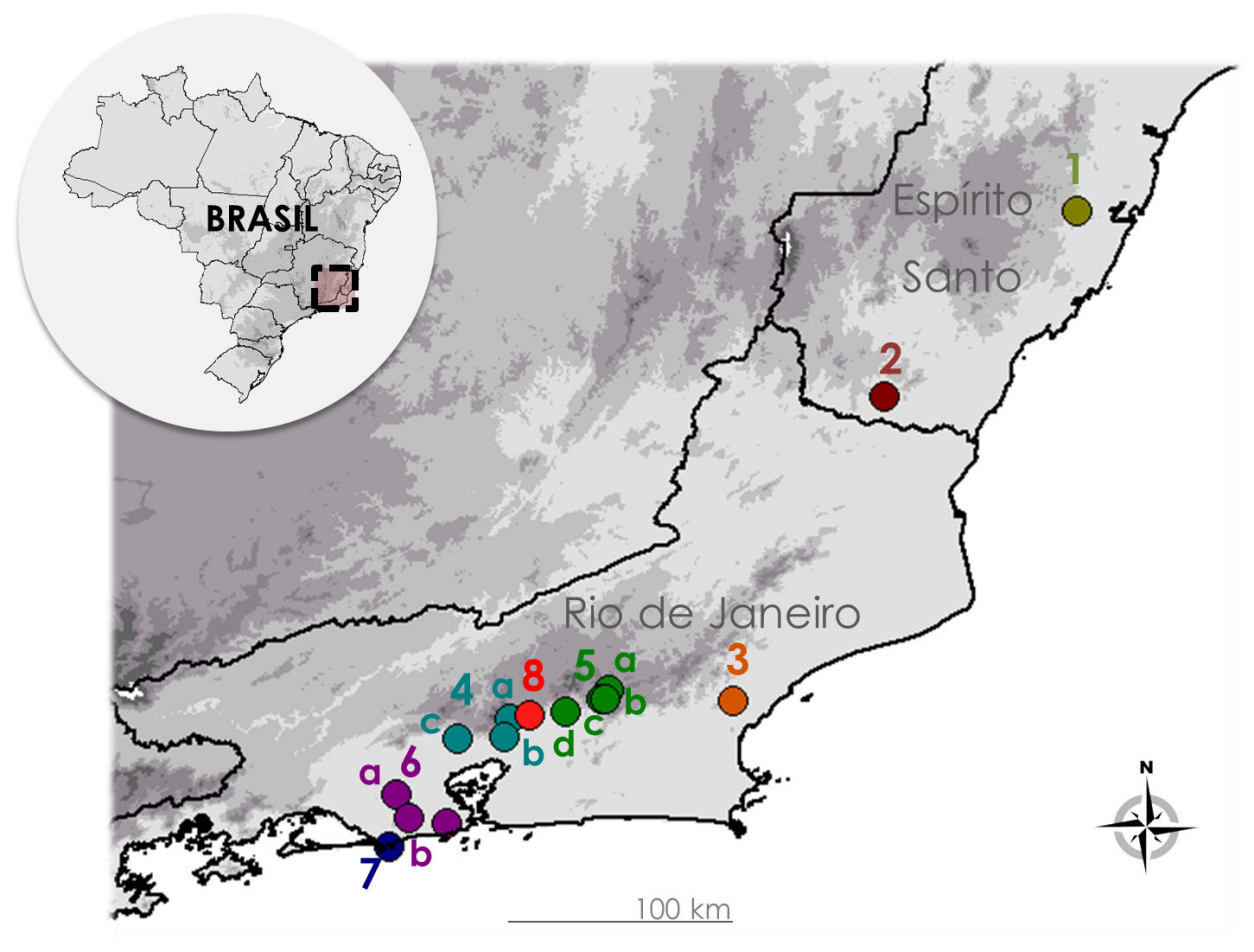
Samples localities of *Euparkerella*. Numbers corresponds to populations of: *Euparkerella tridactyla* (1); *E. robusta* (2); *E. cochranae* (3,4); *E. brasiliensis* (5–7) and *Euparkerella* sp. (8).

We analyzed 39 individuals from all the four known *Euparkerella* species, from 15 localities (28 collected in this study and 11 from scientific collections; Fig. 2; Table S1 in file S1), and one sample from the outgroup species *Barycholos ternetzi* [19]. All field collections did not involve endangered or protected species, and were obtained under appropriate permits (Instituto Chico Mendes de Conservação da Biodiversidade - ICMBio, permit number 18887; and Instituto Estadual do Ambiente - INEA, permit number 040/2010). Techniques used to capture, tissue sampling and euthanasia sought to minimize animal suffering and were in accordance with recommendations of the Herpetological Animal Care and Use Committee (HACC) of the American Society of Ichthyologists and Herpetologists (available at: http://www.asih.org/publications), as well as were accepted by ICMBio and INEA. When collected, individuals were euthanized using an anesthetic application over the skin (5 % Lidocaine), whereas other samples were obtained by toe clipping and followed by specimen release in the field. Prior to toe clipping, a suitable level of anesthetic was applied in the local of incision (2% Lidocaine). 

Following the morphological criteria proposed by Izecksohn [23], we analyzed two specimens of *E. tridactyla*, one of *E. robusta*, 10 of *E. cochranae*, and 22 of *E. brasiliensis*. Since the four individuals from Guapimirim, RJ (Locality 8, see Figure 2 and Table S1 in File S1), were not fully diagnosable as *E. brasiliensis* or *E. cochranae*, we designated them as *Euparkerella* sp. 

### DNA amplification, sequencing and polymorphism

Whole genomic DNA was extracted from tissue samples using the QUIAGEN DNeasy Tissue kit (QUIAGEN). We amplified one mitochondrial gene fragment, cytochrome c oxidase subunit 1 (CO-I), and the nuclear β- fibrinogen intron 7 gene (β-fibint7), a segment with exon 2 and intron 2 of the cellular myelocytomatosis gene (C-myc2), the recombination activating protein 1 gene (RAG-1), and a segment of the exon 1 of tyrosinase gene (TYR). Protocols for DNA extraction, PCR (Polymerase Chain Reaction) gene amplification and sequencing were described in Supporting Information (Protocol S2), and primers used in PCR reactions are listed in Table S2 in File S1 and described in the literature [32–36]. Sequences were edited with the program ChromasPro v. 1.5 (Technelysium Pyt Ltd.). Sequence data were deposited in GenBank and are available under accession numbers KF625052 - KF625166 (Table S1 in File S1), except for β-fibint7 for which amplified sequences were shorter than 200 bp (Table 1), the limit size accepted for this database. Only for this gene, raw sequences are available from the Dryad Digital Repository: http://doi.org/10.5061/dryad.690jk. Alignments were made and manually edited in BioEdit Sequence Alignment Editor [37]. For nuclear genes, the polymorphic nucleotide positions of heterozygous individuals were coded with the IUPAC ambiguity codes. For each heterozygous genotype sequence, we inferred the most likely phased haplotypes with the Bayesian algorithm implemented in PHASE v2.1.1[38]. All known haplotypes were incorporated for haplotype inference. We ran PHASE three times with different random seeds and checked if haplotype estimation was consistent across runs. Each run was conducted using default values. Standard genetic diversity indices were estimated using the program DNAsp v. 5.10.1[39]. Genetic divergence estimated from mtDNA sequences within groups and between pairs of populations were measured by the p-uncorrected distance as implemented in the software MEGA v. 5.1 [40].

**Table 1:** DNA Polymorphism for five loci used in this study.

Fragment	Size (bp)	N	S	h	%Hd (SD)	%π (SD)
**CO-I**	543	34	194	14	92 (2.3)	11 (0.9)
**β-fibint7**	157 - 159	70	24	30	97 (0.6)	3.5 (0.1)
**C-myc2**	458 - 473	70	45	29	94 (1.6)	1.7 (0.1)
**RAG-1**	824	42	38	14	93 (1.5)	0.8 (0.1)
**TYR**	507	42	46	25	97 (1.1)	1.9 (0.2)

N – number of sequences (phased in nuclear genes); S – number of polymorphic positions; h – number of haplotypes; %Hd – haplotype diversity percentage and respective standard deviation (SD); %π nucleotide diversity percentage and respective standard deviation (SD).

### Phylogenetic analysis and divergence time estimates

Phylogenetic analyses were based on independent gene tree inference for the five gene fragments, on traditional multilocus tree inference using sequences from all genes concatenated into a single data matrix, and on the coalescent-based approach implemented in *BEAST, where genes trees are simultaneously estimated to find the best multilocus species tree. The most appropriate model of nucleotide evolution and the best-fitting partitioning scheme were selected using PartitionFinder v. 1.0 [41] under the Akaike information criterion [42] (for adopted models, see Table S3 in File S1). For each partition, we set the number of distinct relative substitution rates and the rate of variation among sites (gamma distribution and/or proportion of invariable sites) that best adjusted to the nucleotide evolution model. The prior on the substitution rate was set as variable to accommodate rate differences across partitions.

The best partition strategy and the most appropriate model of nucleotide evolution (Table S3 in File S1) were used for Bayesian inference with the program MrBayes v.3.1.2 [43], for both gene trees and concatenated multilocus tree estimates. Four MCMC (Markov chain Monte Carlo) were run simultaneously in each analysis, each of one with four chains sampled every 1000 generations, until split frequencies reached 0.002. We removed the first 25% of trees as burn-in and tested convergence of chains plotting log-likelihood values versus number of generations in program Tracer v.1.5 [44]. The species tree was generated in *BEAST [45] implemented in BEAST v.1.7.4 [46]. A priori assignment of individual alleles to a “species”, was based on information from well-supported and geographically structured clades in the multilocus tree (concatenated dataset), the Bayesian population structure analysis, and genealogical indices of population divergence (see the results section). 

To estimate divergence times to the most recent common ancestral (tMRCA) we used the mean nucleotide substitution rates of RAG-1 and TYR obtained by Heinicke et al. [18] for *Terrarana* (0.0015 mutations/site /million years). Although divergence times inferred on the basis of nucleotide substitution rates are not free of drawbacks, and are susceptible to errors due to substitution pattern variability across organisms and genes [47], the lack of independent calibration points (e. g., fossil records, biogeographic events) for the studied group prevents the application of an alternative approach. Although divergence times should be interpreted with caution, we consider the use of mutation rates still valuable to infer a timeframe for hypotheses of historical biogeography and patterns of diversification within *Euparkerella*. Models of nucleotide evolution were simplified with respect to codon positions to avoid overparameterization. Divergence times were estimated using a lognormal relaxed clock. We adopted the Yule speciation process and followed default settings for other parameters. Due to computational limitations, data were analyzed by four independent runs of 50 million generations (total of 200 million) sampled each 5000 generations, and 10% removed as burn-in. We assessed chain convergence with the program Tracer v. 1.5 [44], assembled different runs outputs with LogCombiner v. 1.7.4 [46] (Drummond et al. 2012), and generated the final tree with TreeAnnotator v. 1.7.4 [46]. 

### Delimitation of genetic evolutionary units

In addition to phylogenetic analysis we used several methods to assess taxonomic diversity within the morphology-based species from Rio de Janeiro (*E. brasiliensis*, *E. cochranae*, and *Euparkerella* sp.): 1) cluster assignment analyses based on allele frequencies; 2) Bayesian coalescent-based species delimitation, and; 3) Genealogical Sorting Index. 

Genetic structure analysis were investigated with no a-priori assignment of individuals to clusters using a Bayesian model-based algorithm implemented in the program STRUCTURE v. 2.3.3 [48] and examined independently for nuclear markers only and for mtDNA combined with nuclear markers. Although the use of a Bayesian clustering analysis in our system is not free of drawbacks, especially regarding the low sample size for each of the three entities herein analyzed, which may decrease the power of reliably inferring the optimal number of clusters [49] we still consider the use of such approach as useful for non-phylogenetic discovery tool of species-level diversity and for comparative purposes across different type of methods [50]. Sequence data were converted to Structure input file format using the program xmfa2struct (available at: http://www.xavierdidelot.xtreemhost.com/clonalframe.htm). The STRUCTURE analysis was performed under the admixture ancestry model, with five independent runs for each K ranging from 1 to 10, using 5x10^5^ MCMC repetitions and discarding the first 5x10^4^ iterations as burn-in. To choose the K value that best fitted our data we used the on-line program Structure Harvester v.0.6.93 [51] to monitor the estimated log posterior probability of the data (ln Pr (X/K)) [48], and estimate the second-order rate of change of the likelihood function (ΔK) [49]. We used the program CLUMPP v.1.1.2 [52]. to assemble results of the five independent runs generated by Structure in an average admixture matrix Q, which was represented graphically with the program DISTRUCT v. 1.1 [53].

.Delimitation of putative evolutionary units as inferred by phylogenetic and Bayesian clustering analyses were tested using a multilocus, coalescent-based species delimitation method implemented in the program “Bayesian Phylogenetics and Phylogeography” (BP&P; [54]). This method, that assumes that shared polymorphism is attributable to incomplete sorting of ancestral polymorphism, combines species phylogenies and gene genealogies via ancestral coalescent processes, providing statistical support (posterior probabilities) for different species delimitations. Following most users of BP&P [50,55,56] we used the species tree estimated with *BEAST as guide tree. Since the guide tree may play a critical role in the outcome of the species delimitation model, phylogenetic uncertainties on its topology may constitute an important drawback for accurate species delimitations [50,55]. Given the low nodal support (pp < 0.80) for the relationships among some of the evolutionary lineages recovered by our species tree estimates (*E. cochranae* 3 and *E. brasiliensis* 5, 6 and 7, see results), we followed the recommendation of [55], comparing the results from the use of different guide trees representing all possible phylogenetic resolutions of those evolutionary lineages. So, for the BP&P analysis we considered three different guide trees: (*E. robusta* 2, (*E. tridactyla* 1, ((*Euparkerella* sp. 8 + *E. cochranae* 4), ((*E. brasiliensis* 7 + *E. cochranae* 3), (*E. brasiliensis* 6 + *E. brasiliensis* 5))))); (*E. robusta* 2, (*E. tridactyla* 1, ((*Euparkerella* sp. 8 + *E. cochranae* 4), (*E. cochranae* 3, (*E. brasiliensis* 7, (*E. brasiliensis* 6 + *E. brasiliensis* 5)))))); and (*E. robusta* 2, (*E. tridactyla* 1, ((*Euparkerella* sp. 8 + *E. cochranae* 4), (*E. brasiliensis* 7, (*E. cochranae* 3, (*E. brasiliensis* 6 + *E. brasiliensis* 5)))))). We followed the same methodology used by [55] for adjusts of gamma prior (*G*) in population size (θ) and age of the root in the species tree (τ_0_) parameters. Accordingly, we tested three scenarios to examine the influence of priors in the posterior probabilities: i) assuming relatively large ancestral population sizes and deep divergences (θ = 1, 10, τ_0_ = 1, 10); ii) assuming relatively small ancestral population sizes and shallow divergences among species (θ = 2, 2000, τ_0_ = 2, 2000); and iii) assuming large ancestral population sizes and relatively shallow divergences among species (θ = 2, 2000, τ_0_ = 2, 2000). We ran two rjMCMC analyses for each trial using 500000 generations, 10000 as burn-in, with sample interval of five.

We also used the Genealogical Sorting Index method (gsi) [57] to test the level of genealogical divergence in our nuclear gene trees for each of the evolutionary units inferred by our multilocus phylogenetic analysis and Bayesian clustering analyses within *E. cochranae*, *E. brasiliensis* and *Euparkerella* sp.. This index allows us to take a measure of the degree of exclusive ancestry for single locus gene trees in pre-defined groups [57,58]. To estimate the *gsi*, we first generated rooted trees topologies for each individual nuclear locus. For each, we generated 1000 trees using the Maximum Likelihood (ML) approach implemented in the program RAxMLGUI v.1.2 [59], a graphic interface of RAxML [60], adopting the GTR gamma as model of nucleotide evolution. Subsequently, we generated a Majority Rule consensus tree keeping branches with posterior probabilities higher than 50%. Those 1000 individual *gsi* measurements were used to estimate an ensemble *gsi* statistic (*gsi*_*T*_) for each locus. All values and their significance were obtained at the web server of Genealogical Sorting Index (available in: http://www.genealogicalsorting.org/).

All alignments and main input files used in this work are available from the Dryad Digital Repository: http://doi.org/10.5061/dryad.690jk

## Results

### Sequence variation

Standard genetic diversity summary statistics across loci are summarized in Table 1. For the mtDNA gene (CO-I), we obtained an alignment of 543 base pair sequences, corresponding to 14 unique haplotypes and 194 segregating sites (Table 1). Neither stop nor nonsense codons were detected. Within the genus *Euparkerella*, the pairwise mtDNA sequence divergence ranged from 26.6%, between *E. tridactyla* and *E. robusta* to 6.1 %, between *Euparkerella*. sp. 8 and *E. cochranae* 4; whereas within groups p-distance ranged from 0 % to 2.1 % (Table 2). Within *E. brasiliensis*, the sequence divergence ranged from 6.3% to 11.7%, while within *E. cochranae* this divergence was 13.6% (Table 2).

**Table 2:** Genetic divergence (%) within and among populations, and geographical distance among populations/species of *Euparkerella.*

	**N**	**(1)**	**(2)**	**(3)**	**(4)**	**(5)**	**(6)**	**(7)**	**(8)**
**(1) *E. tridactyla***	2	0.0	125	285	365	320	420	440	450
**(2) *E. robusta***	1	20.63	--	160	240	190	300	315	225
		(±1.73)							
**(3) *E. cochranae***	2	18.78	18.05	0.0	105	60	155	170	90
		(±1.66)	(±1.56)						
**(4) *E. cochranae***	7	19.71	17.22	13.63	2.1	50	60	75	15
		(±1.61)	(±1.54)	(±1.41)					
**(5) *E. brasiliensis***	9	17.82	16.94	9.30	12.48	0.6	105	120	35
		(±1.60)	(±1.51)	(±1.13)	(±1.30)				
**(6) *E. brasiliensis***	6	19.21	16.08	9.64	12.89	6.26	0.5	15	75
		(±1.71)	(±1.49)	(±1.19)	(±1.35)	(±0.95)			
**(7) *E. brasiliensis***	5	20.44	18.78	11.23	14.55	11.23	11.66	0.0	90
		(±1.78)	(±1.66)	(±1.31)	(±1.39)	(±1.33)	(±1.37)		
**(8) *Euparkerella* sp.**	4	20.44	17.68	13.63	6.08	12.38	13.32	14.55	0.0
		(±1.71)	(±1.60)	(±1.46)	(±0.91)	(±1.35)	(±1.42)	(±1.46)	

Genetic divergence estimated for the mitochondrial gene fragment CO-I. Uncorrected *p*-distance (%) and standard error among and within populations/species below and in the the diagonal (underlined), respectively Geographic distance in kilometers above the diagonal. N, number of specimens analyzed.

For nuclear loci, we obtained fragments of 157–159 bp for β-fibint7, 458–473 bp for C-myc2, 824 bp for RAG-1, and 507 for TYR (Table 1). The number of haplotypes ranged from 14 for RAG-1, to 30 for β-fibint7 (Table 1).

### Gene trees

**Figure 3:**
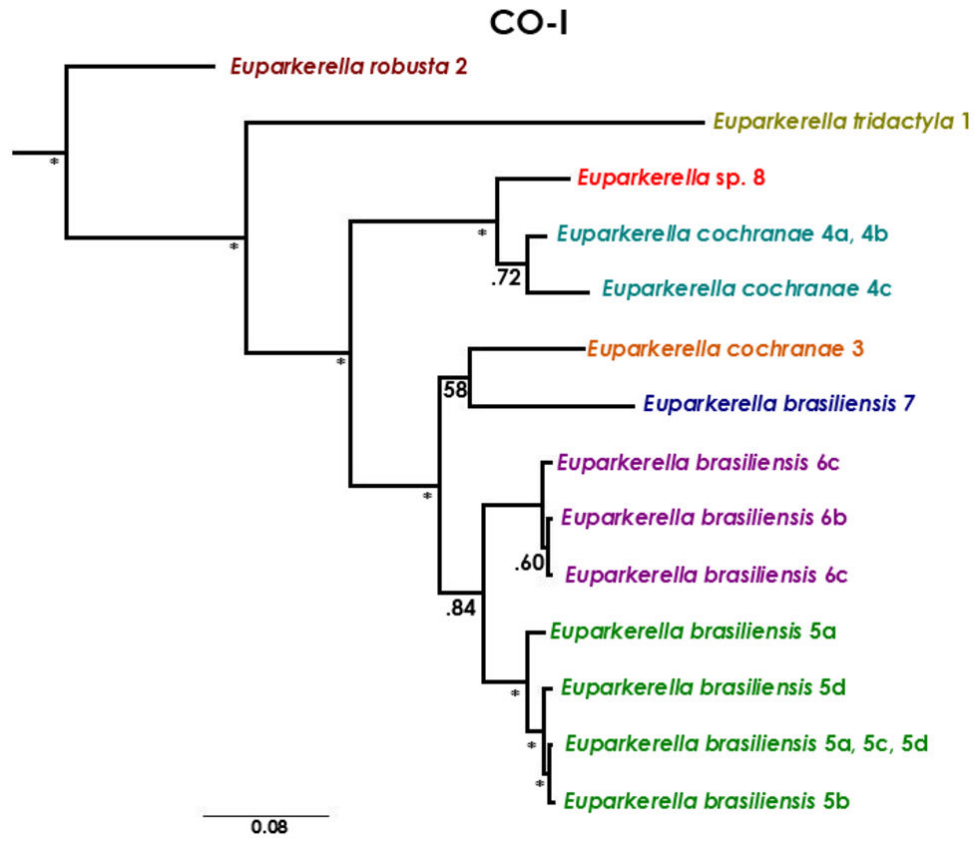
Mitochondrial (CO-I) gene tree of *Euparkerella*. Bayesian phylogenetic inference of mitochondrial haplotypes of *Euparkerella*. Colors refer to populations and combinations of numbers-letters indicate localities (Figure 2 and Table S1 in File S1) corresponding to haplotypes. Posterior probabilities are indicated to the left of nodes. Asterisks represents posteriors equal or higher than 0.95.

**Figure 4:**
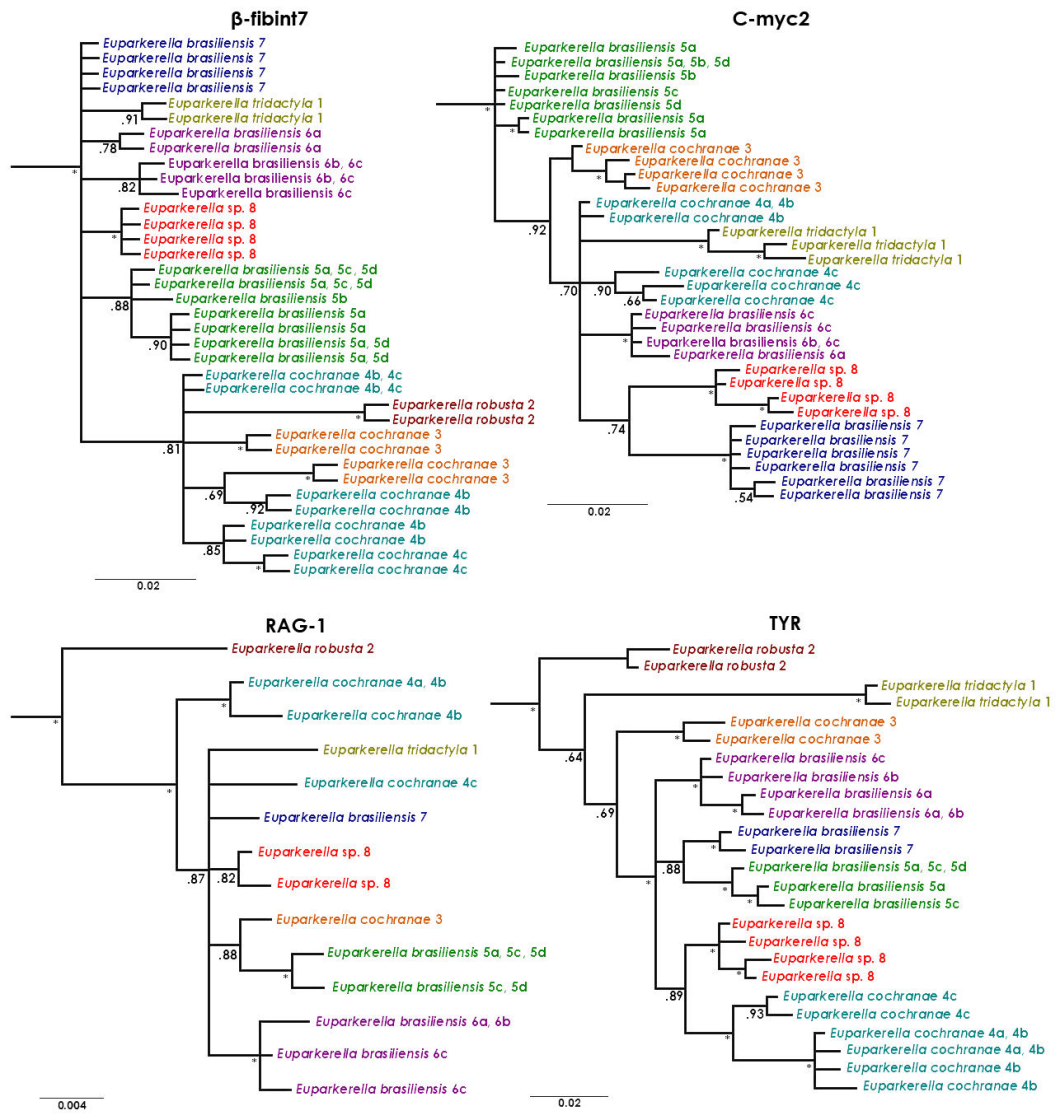
Nuclear genes trees of *Euparkerella*. Bayesian phylogenetic inferences of nuclear haplotypes of four gene fragments (β-fibint7, C-myc, RAG-1 and TYR) of *Euparkerella*. Colors refer to populations and combinations of numbers-letters indicate localities (Figure 2 and Table S1 in File S1) corresponding to haplotypes. Posterior probabilities are indicated left to nodes. Asterisks represents posteriors equal or higher than 0.95.

The Bayesian inference of the mtDNA CO-I gene tree resulted in an overall highly resolved and strongly supported topology for all major relationships (Figure 3). Contrasting with the previously morphologically-defined species *E. robusta* and *E. tridactyla*, both *E. brasiliensis* and *E. cochranae* were not recovered as monophyletic groups. *Euparkerella cochranae* 3 (from locality 3) and *E. brasiliensis* (from localities 5, 6 and 7) form a well-supported clade, and *E. cochranae* 4 (from locality 4) together with individuals belonging to *Euparkerella* sp. (locality 8) form another clade with high posterior probability. The former clade appears to be highly substructured with the occurrence of two sub-clades with high posterior probability, corresponding to individuals of *E. brasiliensis* from localities 5 and 6, but their relationships are basically unresolved (Figure 3). Although resolution varied among nuclear loci, gene trees showed an overall lower resolution when compared to the mtDNA tree, and some phylogenetic non-concordance (Figure 4). The placement of *E. cochranae* 3 was non-concordant across markers, by clustering with *E. cochranae* 4 and *E. robusta* on the β-fibint7 tree, but with its position unresolved for other nuclear markers. Another non-concordant pattern between mtDNA and nuclear gene trees was the monophyly of *Euparkerella* sp., which was recovered by all nuclear, but not mtDNA, gene trees (Figure 4).

### Multigene trees and divergence time estimates

**Figure 5:**
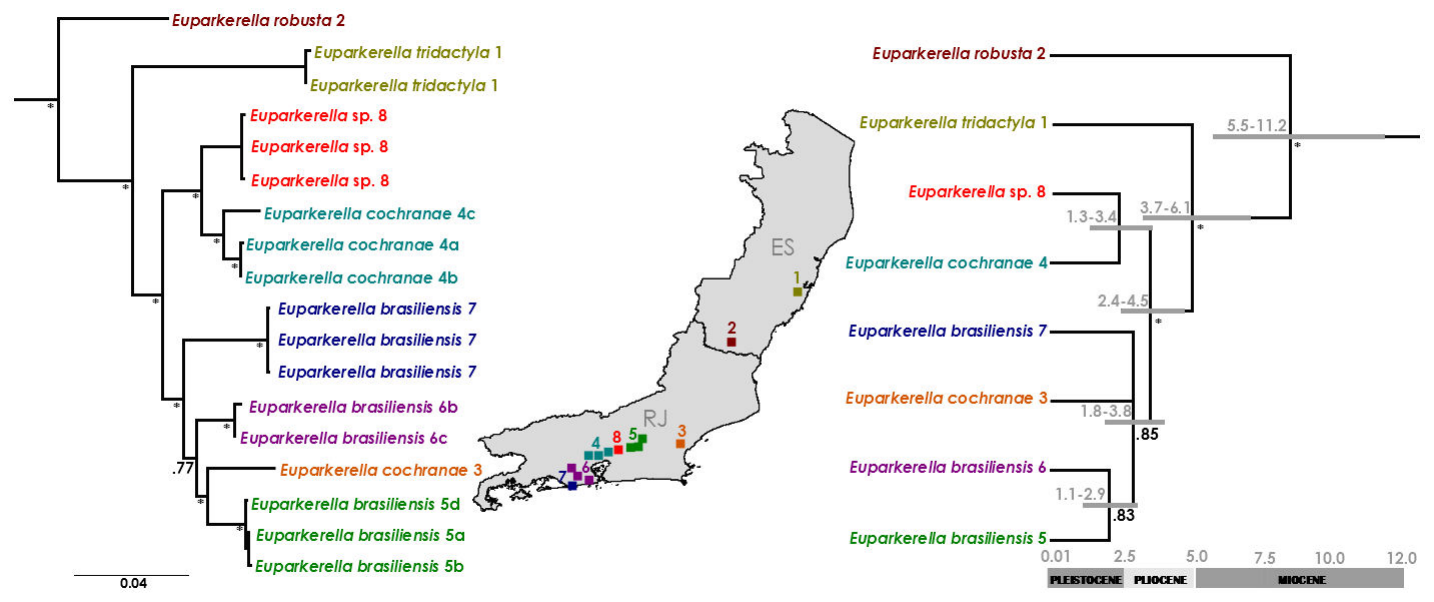
Multilocus trees of *Euparkerella*. Bayesian phylogenetic inference of concatenated sequence data (left) and *BEAST Species Tree (right) of *Euparkerella*. Colors refer to populations indicated on the map (see Table S1 in File S1 for details). Posterior probabilities are indicated near nodes. Asterisks represent posteriors equal or higher than 0.95. Grey bars in Species Tree indicate 95% interval of tMRCA estimated in million years.

Both the multilocus tree resulting from concatenated data analysis and the species tree showed very similar topologies, but with better resolution and higher level of node support for the concatenated multilocus tree (Figure 5). Both *E. brasiliensis* and *E. cochranae* grouped in two different clades, a pattern also seen on the mtDNA tree. However, only the mtDNA and the concatenated multilocus tree showed high posterior probability for the clade formed by all *E. brasiliensis* individuals (localities 5, 6 and 7) and *E. cochranae* 3. Estimates of tMRCA indicated that the diversification of *Euparkerella* started in Miocene (11.2 and 5.5 million years ago). Subsequent diversifications in the genus appear to have occurred in the Pliocene and at the Plio-Pleistocene boundary (Figure 5).

### Delimitation of evolutionary units

**Figure 6:**
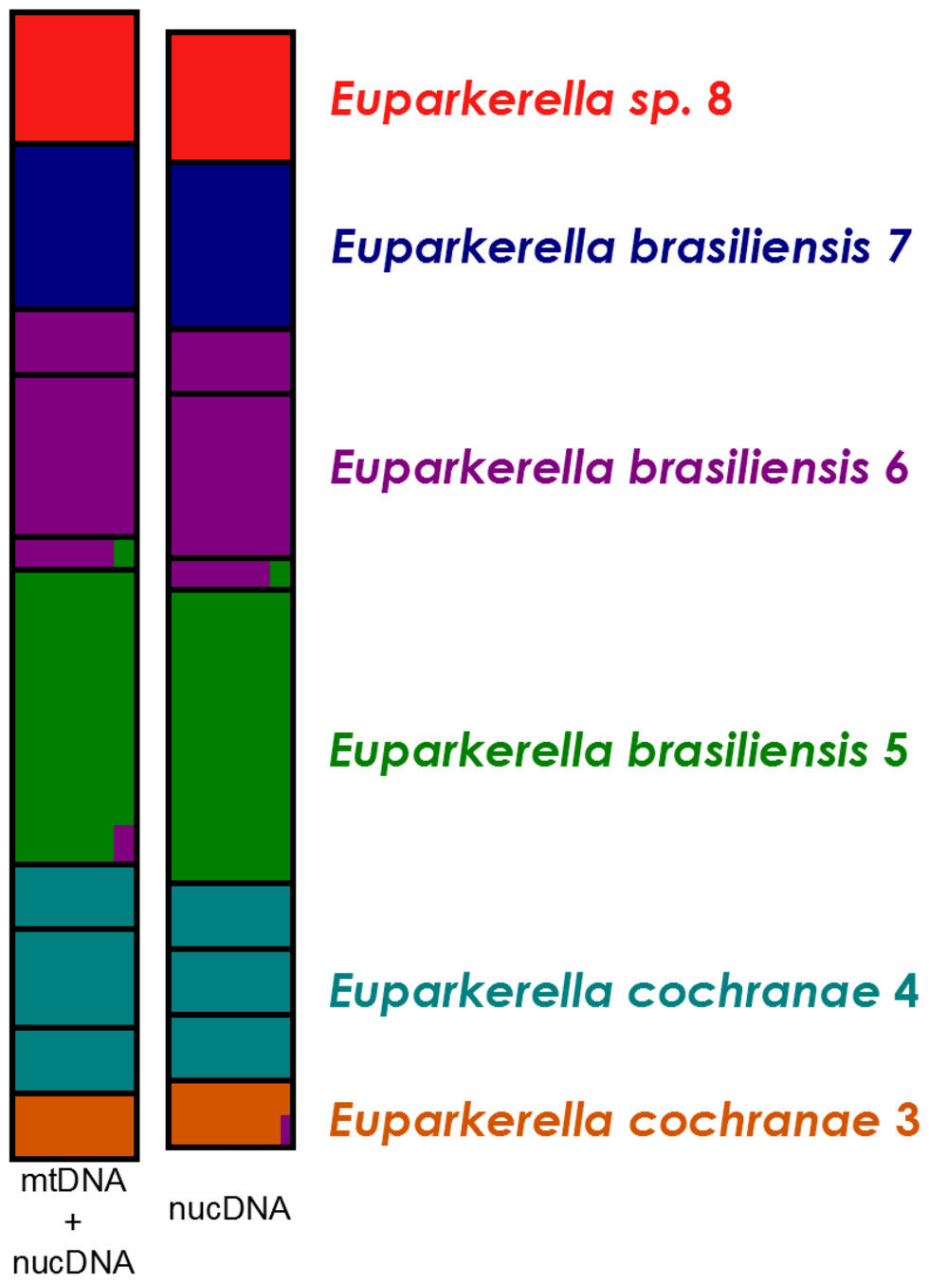
Structure results for *Euparkerella* populations. Assignment proportion of individuals to populations of *Euparkerella* from Rio de Janeiro State. Structure results for number of groups K=6. Left bar includes both mitochondrial and nuclear genes, right bar includes only nuclear genes.

The level of genetic structure recovered by the Bayesian clustering methods for both nuclear and combined nuclear and mtDNA datasets of *E. brasiliensis, E. cochranae*, and *Euparkerella* sp. was *K* = 6, as suggested by both the highest log posterior probability of the data (ln Pr (X/K) and the ΔK score (Figure 6). Each of the six sampled localities of Rio de Janeiro was classified into distinct genetic clusters with high individual membership coefficient (0.95 to 1.0). Moreover, there is an overall concordance between these six distinct clusters with terminal clades inferred by phylogenetic analyses. 

**Figure 7:**
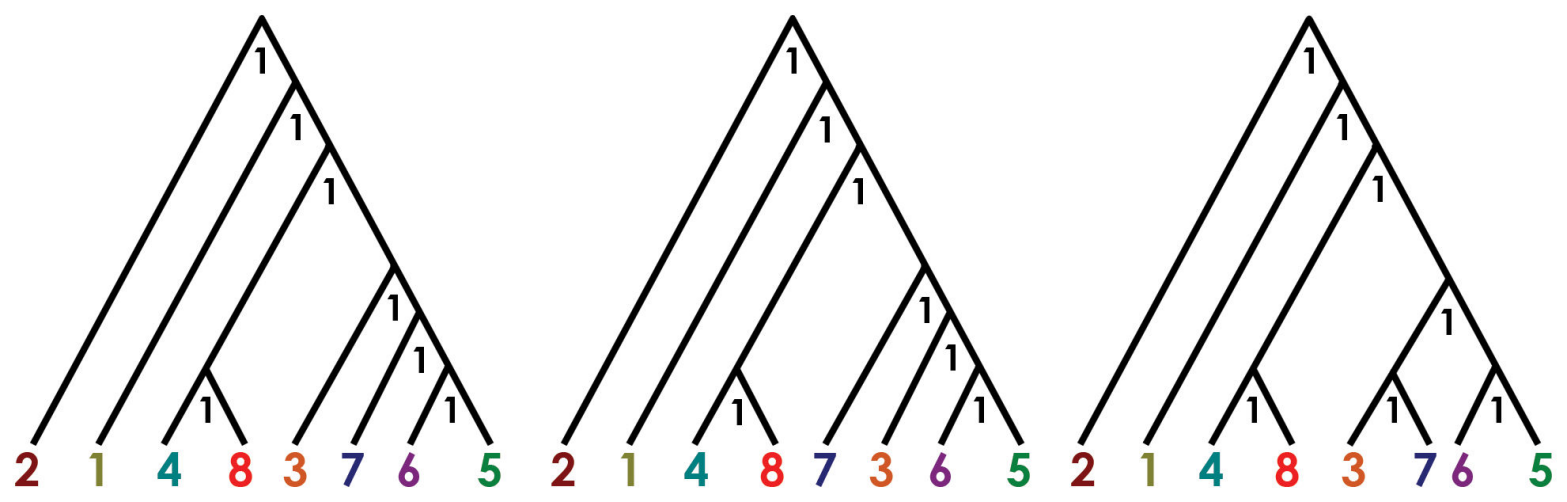
BP&P species delimitation. Bayesian species delimitation assuming three alternative phylogenetic arrangement based in *BEAST species tree (see Figure 5). Numbers below the nodes represent speciation probabilities values. Colors and numbers refers to populations indicated on the map (Figure 2, see Table S1 in File S1 for details).

The Bayesian species delimitation using BPP analyses are shown in Figure 7. BPP analyses produced speciation probabilities of 1.0 on all nodes across all three combinations of prior distributions for θ and τ_0_ and alternative guide trees (Figure 7). Estimates of genealogical sorting index (gsi) were highly significant (all p-values < 0.02) for each nuclear locus across all six evolutionary units as defined by phylogenetic inferences and Bayesian clustering analysis, and across loci within those units, ranging from 0.55 to 1.0, and from 0.89 to 1.0, respectively (Table 3). 

**Table 3:** Genealogical sorting index for *Euparkerella* populations/species.

	***E. cochranae***		***E. cochranae***		***E. brasiliensis***		***E. brasiliensis***		***E. brasiliensis***		***Euparkerella* sp.**	
	**(3)**		**(4)**		**(5)**		**(6)**		**(7)**		**(8)**	
	*gsi*	*P*	*gsi*	*P*	*gsi*	*P*	*gsi*	*P*	*gsi*	*P*	*gsi*	*P*
**β-fibint7**	0,5803	0,0001	0,8327	0,0001	1	0,0001	0,7647	0,0001	1	0,0001	1	0,0001
**C-myc2**	1	0,0001	1	0,0001	0,8582	0,0001	1	0,0001	1	0,0001	1	0,0001
**RAG-1**	1	0,0152	0,5486	0,0001	1	0,0001	1	0,0001	1	0,0001	1	0,0002
**TYR**	1	0,017	1	0,0001	1	0,0002	1	0,0001	1	0,0001	1	0,0001
**total (*gsi*_*T*_)**	0,8951	0,0001	0,8453	0,0001	0,9646	0,0001	0,9412	0,0001	1	0,0001	1	0,0001

Genealogical sorting index (gsi) and respective probabilities (*P*) estimated for each nuclear gene and total (*gsi*_*T*_) for groups of *Euparkerella* from Rio de Janeiro State.

## Discussion

The first study examining the genetic diversity within the genus *Euparkerella* uncovered high levels of genetic diversity within the two morphological species *E. cochranae* and *E. brasiliensis*, which is in line with previous studies of *Terrarana* [5,11,12,61]. The gene trees at five independent loci and trees from combined data (concatenated dataset and the species tree) allowed us to uncover at least six deeply diverged and geographically coherent evolutionary units: two within *E. cochranae*, three within *E. brasiliensis*, and another corresponding to *Euparkerella* sp., which may have diversified between the Miocene and the Pleistocene. The delimitation of these six units was supported by all methods herein used, including Bayesian clustering analysis, Bayesian coalescent-based species delimitation, and the Genealogical sorting index, providing thus strong evidence for a current underestimation of diversity in *Euparkerella*. 

### Phylogenetic analyses

Topologies of nuclear DNA gene trees were somewhat discordant, revealing less power than mitochondrial DNA (CO-I) in resolving phylogenetic relationships. Discordance between nuclear and mitochondrial markers is expected in analyses of closely related species [62,63]. Our results notably showed however the concordance between mtDNA and most nDNA loci in recovering of several clades within *E. cochranae* (two clades) and *E. brasiliensis* (three clades). The mtDNA CO-I tree additionally showed high support for four main groups that correspond to *E. robusta* and *E. tridactyla*, which are basal relative to other two well supported sister clades: one composed by *E. cochranae* 4 and *Euparkerella* sp. 8, and the other including *E. brasiliensis* 5, *E. brasiliensis* 6*, E. brasiliensis* 7, and *E. cochranae* 3.

Phylogenetic relationships within *Euparkerella* are better supported in the tree inferred using the multilocus concatenated dataset when compared either to the mtDNA tree or the multilocus species tree, as observed in several other case studies [30,64–67]. This is likely explained by differences in the sensitivity of distinct methods to gene tree incongruences. While species trees are more sensitive to the resolution of independent gene trees, reconstructions from concatenated datasets enable that lesser resolution given by a particular gene may be compensated by the higher resolution of another gene [45,67,68]. The higher resolution of the CO-I data would compensate the lesser resolution of the nuclear loci in the tree inferred from concatenated data, and incongruences between the several loci would decrease the resolution of the species tree. This appears to be exactly the case of clade *E. cochranae* 3 - *E. brasiliensis*, which shows distinct degrees of resolution depending on the tree reconstruction method (well resolved for the CO-I and concatenated datasets, and a polytomy for the species tree). Thus, we suggest that the conflict between the tree inferred from concatenated data and the species tree is explained by gene tree incongruences affecting the latter and not necessarily by a wrong tree inference from the concatenated dataset [69]. The degree of population and gene sampling undertaken in this initial study of genetic diversification within *Euparkerella* limits our ability to discuss this matter any further. Future work sampling more individuals and localities of all species of *Euparkerella*, and covering more of the genome diversity perhaps will result in a more complete understanding of phylogenetic relationships in this genus.

### Species delimitation: diversification within the genus *Euparkerella*

The combined nuclear and cytoplasmic DNA data uncovered several genetic units in *Euparkerella*, especially within the previously recognized species *E. cochranae* and *E. brasiliensis*. There is general concordance between gene trees and trees from combined data in recovering two clades within *E. cochranae* (*E. cochranae* 3 and *E. cochranae* 4), three clades within *E. brasiliensis* (*E. brasiliensis* 5, *E. brasiliensis* 6, and *E. brasiliensis* 7), and *Euparkerella* sp., all corresponding to specific localities or regions. Population structure inferred from Bayesian analysis of multilocus nuclear data, and from combined cytoplasmic and nuclear data, suggests six genetically differentiated units that correspond to the six clades recovered from phylogenetic analyses. Each geographic region corresponds to a distinct genetic group with probabilities between 0.95 and 1.0. This overall congruence between results from both phylogenetic and Bayesian clustering methods give additional confidence for the delimitation of six distinct evolutionary units within the three previously defined *Euparkerella* species. So, despite the limitations of our data (reduced sample size and meaningful species-level genetic diversity), this result is in line with the observed in other studies that used Bayesian clustering analysis to identify and/or delimit evolutionary units, even within widespread species complexes [56,58,70] 

The Bayesian species delimitation method (BPP) consistently supported the same six distinct evolutionary units as inferred by both phylogenetic and Bayesian clustering analysis, even accounting for variation on population sizes and divergence times. The BPP analysis may have the drawback of relying on an accurate guide tree [55] which in our case (*BEAST species tree) had some ambiguous relationships estimates. Previous studies have indeed shown that BPP can produce misleading results due to incorrectly prior-specified trees [50,55,71]. However, *Euparkerella* species delimitation using BPP consistently recovered posterior probabilities of 1.0 for every speciation event when we used different guide trees representing all possible branching patterns in regions of the species tree with low support. Moreover, using an independent method (GSI), these six genetic evolutionary units are characterized by high levels of genealogical exclusivity both for individual nuclear gene trees (*gsi*) and in combined multilocus analysis (*gsi*_*T*_) (Table 3), which is consistent with a history of independent divergence for these units [57,58,72].

Whether our genetic units conform or not to a particular species concept is less relevant than the processes of old evolutionary diversification uncovered for *E. brasiliensis* and *E. cochranae*. The observed pairwise mtDNA genetic divergences among the three units (clades) of *E. brasiliensis* (6,3% - 11,2%) and between the two units (clades) of *E. cochranae* (13,6%) are generally similar or larger than genetic divergences observed among other amphibian species [4,25,73,74]. Divergence times inferred from the *Euparkerella* species tree suggest that diversification of those genetic units occurred between the Miocene and the Plio-Pleistocene boundary (1.4 - 5.1 mybp), which is also concordant to times of divergence inferred for other species of amphibians [27,34], including species of *Terrarana* [12].

### Geographical patterns of genetic diversity

High levels of genetic substructure were observed within *Euparkerella*, with all know genetic units occurring in single localities or geographically restricted locality groups, without any records of sympatry. The six genetic units uncovered within *Euparkerella* from Rio de Janeiro State correspond to populations that are between 15 and 100 km away from the closest congeneric populations. Diversification in relatively small geographic scales seems relatively common in small-bodied, and particularly in miniaturized vertebrates [25,27,30,75,76]. Organismal body size has several functional, ecological, and evolutionary implications, in particular reducing physiological tolerance (e.g., higher sensitivity to dehydration) and dispersal in the case of extremely small-bodied species [27,77]. The lower levels of gene flow between populations induced by those morphophysiological traits may lead to range fragmentation at relatively small geographic scales and posterior genetic diversification, even without apparent barriers to gene flow [27]. We hypothesize the same general processes in small-bodied or miniaturized species determined the geographical structure and divergence observed in *E. brasiliensis* and *E. cochranae*, perhaps combined with habitat heterogeneity. Within the range of *Euparkerella* in Rio de Janeiro, the landscape is formed by mountains that promote altitudinal climatic variation (from sea level to 1000 meters) accompanied by considerable physiognomic diversity (Restinga - a coastal vegetation with marine influence; Lowland rainforest, 5–50m a.s.l..; Submontane rainforest, 50–500m a.s.l.; and Montane rainforest, 500–1500m a.s.l.) [78]. The three genetic units recovered within *E. brasiliensis* occur in three distinct physiognomies: *E. brasiliensis* 7 occurs in the Restinga, whereas *E. brasiliensis* 5 and 6 occur in Submontane and Montane rainforest. Our results are indicative of some association between small scale habitat heterogeneity and the diversification of *Euparkerella*. This study cannot rule out factors that are commonly mentioned as promoters of geographic isolation, such as geographic barriers, climate change, and other sources of habitat disturbance [79–82], to have been involved in the diversification of *Euparkerella*. Alternative hypotheses explaining diversification in *Euparkerella* will only be properly examined when studies combining higher geographic sampling with analyses of physiological, behavioral, ecological, and environmental variables are undertaken in the future.

### Taxonomical and conservation implications

The present study was the first to examine genetic diversity within the genus *Euparkerella*. Genetic diversity was very high and six distinct genetic units have likely independently diversified since the late Pliocene. Our results imply in some cases that morphological traits previously used to distinguish species are not truly diagnostic. For example, *E. cochranae* 3 is morphologically indistinguishable from *E. cochranae* 4 (the two are currently recognized as the same species), whereas both are not sister clades in our phylogenetic analyses. Discordance between molecular and morphological data in diagnosing small-bodied or miniaturized species is relatively common and may be associated to the reduction of visible characters [75]. One way to overcome this limitation will be to undertake detailed morphological, anatomical, and osteological analyses using high resolution techniques, such as geometrical morphometry, radiometric imaging and high-contrast 3D imaging throughout X-ray micro and nanotomography [25,30,83]. An integrative analysis of morphology, physiology, behavior, and ecology will likely be instrumental in the future to properly undertake a much needed taxonomic revision of the genus *Euparkerella*. 

Taken together, the high levels of genetic diversity uncovered within *Euparkerella* at small geographic scales suggest that the spatial scale of sampling may be essential to find cryptic diversity in reduced size amphibians. Given the intrinsic morphoecological characteristics of *Euparkerella* combined with habitat heterogeneity it is likely that genetic units in this genus may increase with higher sampling, especially across the ranges of the species *E. tridactyla* and *E. robusta*. We predict that such levels of cryptic diversity, whether corresponding to incipient or fully-fledged speciation, may be excellent models to examine the origins and maintenance of microendemism in the context of spatial heterogeneity and/or human induced fragmentation of the highly threatened Brazilian Atlantic forest hotspot.

## Supporting Information

Supporting Information Tables S1, S2, S3 (DOCX)

Protocol S1 (DOCX)
