# Supplementary material for: Cryptic Genetic Diversity Is Paramount in Small-Bodied Amphibians of the Genus Euparkerella (Anura: Craugastoridae) Endemic to the Brazilian Atlantic Forest
Source: PLoS One. 2013 Nov 1;8(11):e79504. doi: 10.1371/journal.pone.0079504 (PMC3815154; doi:10.1371/journal.pone.0079504)
Supplement: Supporting Information Tables S1, S2, S3 — (DOCX) [file pone.0079504.s001.docx]

**Table S1. Localities and samples included in molecular phylogenetic analysis.**

| **Species** | **Locality** | **Decimal Degrees** | | **Altitude** | **Voucher** | **GenBank Accession Nº** | | | |
| --- | --- | --- | --- | --- | --- | --- | --- | --- | --- |
|  |  | **Latitude** | **Longitude** | **(m)** | **Nº** | **CO-I** | **Cmyc-2** | **RAG** | **TYR** |
| *Euparkerella tridactyla* | Cariacica, ES **(1)** | -20.281111 | -40.521944 | 550 | JFT305 | KF625052 | KF625131 | KF625087 | KF625109 |
| *Euparkerella tridactyla* | Cariacica, ES **(1)** | -20.281111 | -40.521944 | 550 | JFT346 | KF625053 | KF625132 | KF625088 | KF625110 |
| *Euparkerella robusta* | Mimoso do Sul, ES **(2)** | -21.092142 | -41.363772 | 106 | MNRJ60995 | KF625054 | - | KF625089 | KF625111 |
| *Euparkerella cochranae* | Rio das Ostras, RJ **(3)** | -22.421472 | -42.025528 | 41 | **MNRJ86223** | KF625055 | KF625133 | - | **-** |
| *Euparkerella cochranae* | Rio das Ostras, RJ **(3)** | -22.421472 | -42.025528 | 41 | **LAF-X** | KF625056 | KF625134 | KF625090 | KF625112 |
| *Euparkerella cochranae* | Guapimirim, RJ **(4a)** | -22.500000 | -43.000000 | 457 | MNRJ51357 | KF625057 | KF625135 | - | - |
| *Euparkerella cochranae* | Guapimirim, RJ **(4a)** | -22.500000 | -43.000000 | 457 | MNRJ51358 | KF625058 | - | KF625091 | KF625113 |
| *Euparkerella cochranae* | Magé, RJ **(4b)** | -22.577778 | -43.029167 | 44 | MNRJ56146 | KF625059 | KF625136 | KF625092 | KF625114 |
| *Euparkerella cochranae* | Magé, RJ **(4b)** | -22.577778 | -43.029167 | 44 | **MNRJ86260** | KF625060 | **-** | KF625093 | KF625115 |
| *Euparkerella cochranae* | Magé, RJ **(4b)** | -22.577778 | -43.029167 | 44 | **MNRJ86262** | KF625061 | KF625137 | **-** | **-** |
| *Euparkerella cochranae* | Duque de Caxias, RJ **(4c)** | -22.587222 | -43.233164 | 370 | MNRJ72082 | KF625062 | KF625138 | KF625094 | KF625116 |
| *Euparkerella cochranae* | Duque de Caxias, RJ **(4c)** | -22.587222 | -43.233164 | 370 | MNRJ72083 | KF625063 | KF625139 | - | - |
| *Euparkerella brasiliensis* | Nova Friburgo, RJ **(5a)** | - 22.372113 | - 42.570088 | 982 | **MNRJ86228** | KF625064 | KF625140 | **-** | **-** |
| *Euparkerella brasiliensis* | Nova Friburgo, RJ **(5a)** | - 22.372113 | - 42.570088 | 982 | **MNRJ86229** | KF625065 | KF625141 | KF625095 | KF625117 |
| *Euparkerella brasiliensis* | Nova Friburgo, RJ **(5a)** | - 22.372113 | - 42.570088 | 982 | **MNRJ86230** | KF625066 | KF625142 | **-** | **-** |
| *Euparkerella brasiliensis* | Nova Friburgo, RJ **(5a)** | - 22.372113 | - 42.570088 | 982 | **MNRJ86231** | KF625067 | KF625143 | **-** | **-** |
| *Euparkerella brasiliensis* | Nova Friburgo, RJ **(5a)** | - 22.372113 | - 42.570088 | 982 | **MNRJ86232** | KF625068 | KF625144 | **-** | **-** |
| *Euparkerella brasiliensis* | Nova Friburgo, RJ **(5b)** | -22.417358 | -42.604267 | 450 | **MNRJ86239** | KF625069 | KF625145 | KF625096 | KF625118 |
| *Euparkerella brasiliensis* | Cachoeiras de Macacu, RJ **(5c)** | -22.416667 | -42.583333 | 600 | MNRJ56927 | KF625070 | KF625146 | - | - |
| *Euparkerella brasiliensis* | Cachoeiras de Macacu, RJ **(5d)** | -22.466881 | -42.757014 | 70 | **TP54*** | KF625071 | KF625147 | KF625097 | KF625119 |
| *Euparkerella brasiliensis* | Cachoeiras de Macacu, RJ **(5d)** | -22.466881 | -42.757014 | 70 | **TP287*** | KF625072 | KF625148 | **-** | **-** |
| *Euparkerella brasiliensis* | Rio de Janeiro, RJ **(6a)** | -22.834028 | -43.49725 | 94 | MNRJ58154 | - | KF625149 | KF625098 | KF625120 |
| *Euparkerella brasiliensis* | Rio de Janeiro, RJ **(6b)** | -22.932431 | -43.442536 | 102 | **MNRJ86258** | KF625073 | KF625150 | KF625099 | KF625121 |
| *Euparkerella brasiliensis* | Rio de Janeiro, RJ **(6b)** | -22.932431 | -43.442536 | 102 | **MNRJ86259** | **-** | KF625151 | KF625100 | KF625122 |
| *Euparkerella brasiliensis* | Rio de Janeiro, RJ **(6c)** | -22.958223 | -43.278287 | 405 | **MNRJ86250** | **-** | KF625152 | **-** | **-** |
| *Euparkerella brasiliensis* | Rio de Janeiro, RJ **(6c)** | -22.958223 | -43.278287 | 405 | **MNRJ86251** | **-** | KF625153 | **-** | **-** |
| *Euparkerella brasiliensis* | Rio de Janeiro, RJ **(6c)** | -22.958223 | -43.278287 | 405 | **MNRJ86252** | KF625074 | KF625154 | KF625101 | KF625123 |
| *Euparkerella brasiliensis* | Rio de Janeiro, RJ **(6c)** | -22.958223 | -43.278287 | 405 | **MNRJ86253** | KF625075 | KF625155 | **-** | **-** |
| *Euparkerella brasiliensis* | Rio de Janeiro, RJ **(6c)** | -22.958223 | -43.278287 | 405 | **MNRJ86257** | KF625076 | KF625156 | **-** | **-** |
| *Euparkerella brasiliensis* | Rio de Janeiro, RJ **(7)** | -23.057528 | -43.534389 | 10 | GRU26 | KF625077 | KF625157 | KF625102 | KF625124 |
| *Euparkerella brasiliensis* | Rio de Janeiro, RJ **(7)** | -23.057528 | -43.534389 | 10 | MNRJ86224 | KF625078 | KF625158 | KF625103 | KF625125 |
| *Euparkerella brasiliensis* | Rio de Janeiro, RJ **(7)** | -23.057528 | -43.534389 | 10 | MNRJ86225 | KF625079 | KF625159 | KF625104 | KF625126 |
| *Euparkerella brasiliensis* | Rio de Janeiro, RJ **(7)** | -23.057528 | -43.534389 | 10 | MNRJ86226 | KF625080 | KF625160 | - | - |
| *Euparkerella brasiliensis* | Rio de Janeiro, RJ **(7)** | -23.057528 | -43.534389 | 10 | MNRJ59067 | KF625081 | KF625161 | - | - |
| *Euparkerella sp.* | Guapimirim, RJ **(8)** | -22.488302 | -42.919494 | 195 | **MNRJ86220** | KF625082 | KF625162 | KF625105 | KF625127 |
| *Euparkerella sp.* | Guapimirim, RJ **(8)** | -22.488302 | -42.919494 | 195 | **MNRJ86221** | KF625083 | KF625163 | **-** | **-** |
| *Euparkerella sp.* | Guapimirim, RJ **(8)** | -22.488302 | -42.919494 | 195 | **MNRJ86222** | KF625084 | KF625164 | KF625106 | KF625128 |
| *Euparkerella sp.* | Guapimirim, RJ **(8)** | -22.488302 | -42.919494 | 195 | **LAF-IX** | KF625085 | KF625165 | KF625107 | KF625129 |
| *Barycholos ternetzi* | Gurupi, TO | -11.810861 | -48.96975 | - | CFBH23511 | KF625086 | KF625166 | KF625108 | KF625130 |

Localities are numerated according to de map (see Figure 1). Voucher numbers in bold letters indicates specimens collected by us in this study. Asterisks mark sample identification of tissues collected by toe clipping, without voucher. Collections: MNRJ - Laboratório de Herpetologia do Museu Nacional da Universidade Federal do Rio de Janeiro; CFBH - Célio F. B. Haddad, Instituto de Biociências da Universidade Estadual Paulista Júlio Mesquita Filho. JFT – Collection of Animal Tissues of Departamento de Ciências Biológicas, Universidade Federal do Espírito Santo, identified with personal field number of João Filipe Tonini. GRU – Personal field number of Felipe B. S. Telles. LAF – Personal field collection number of Luciana A. Fusinatto (specimens not yet deposited in scientific collection).

**Table S2**. **Primers used in Polymerase Chain Reactions.**

| **Fragment** | **Primers** | **Sequence** | **Reaction** | **Origin** |
| --- | --- | --- | --- | --- |
| **CO-I** | AnW | 5’-AGA CCA ARR GCC TTC AAA G-3’ | - | Mariana Lyra (unpublished) |
|  | AnCOIR | 5’-CCA AAG AAT CAR AAD AAG TGT TG-3’ | - | Mariana Lyra (unpublished) |
| **β-fibint7** | FibX7 | 5’-GGA GAN AAC AGN ACN ATG ACA ATN CAC-3’ | 1ª | [32] |
|  | FibX8 | 5’-ATC TNC CAT TAG GNT TGG CTG CAT GGC-3’ | 1ª | [32] |
|  | BFXF | 5’-CAG YAC TTT YGA YAG AGA CAA YGA TGG-3’ | 2ª | [32] |
|  | BFXR | 5’-TTG TAC CAC CAK CCA CCA CCR TCT TC-3’ | 2ª | [32] |
| **C-myc2** | Cmyc1U | 5’-GAG GAC ATC TGG AAR AAR TT-3’ | 1ª | [33] |
|  | Cmyc3cat | 5’-GTTGYTGCTG ATCTGTTTGAG-3’ | 1ª e 2ª | [34] |
|  | CmycF | 5’-ATA- GGAACCTGTAGGACCAG-3’ | 2ª | [34] |
| **RAG-1** | MartFL1 | 5’-AGC TGG AGY CAR TAY CAY AAR ATG-3’ | 1ª | [35] |
|  | MarR6 | 5’-GTG TAG AGC CAR TGR TGY TT-3’ | 1ª | [35] |
|  | AmpF2 | 5’-ACN GGN MGI CAR ATC TTY CAR CC-3’ | 2ª | [35] |
|  | AmpR1 | 5’-AAC TAC GCT GCA TTK CCA ATR TCA CA-3’ | 2ª | [35] |
| **TYR** | Tyr 1B | 5’-AGG TCC TCY TRA GGA AGG AAT G-3’ | 1ª | [36] |
|  | Tyr 1C | 5’-GGC AGA GGA WCR TGC CAA GAT GT-3’ | 2ª | [36] |
|  | Tyr 1E | 5’-GAG AAG AAA GAW GCT GGG CTG AG-3’ | 1ª | [36] |
|  | Tyr 1G | 5’-TGC TGG GCR TCT CTC CAR TCC CA-3’ | 2ª | [36] |

First or second reactions are indicated for nested Polymerase Chain Reactions.

**Table S3. Models of nucleotide evolution adopted for each gene (or codon position) used in molecular phylogenetic analysis of *Euparkerella*.**

| Fragment | Models |
| --- | --- |
| **CO-I** |  |
| 1^st^ position | K80+I+G |
| 2^nd^ position | GTR |
| 3^rd^ position | GTR+G |
| **β-fibint7** | GTR+G |
| **C-myc2** | GTR+G |
| **RAG-1** | HKY+G |
| **TYR** |  |
| 1^st^ e 2^nd^ positions | HKY+I |
| 3^rd^ position | HKY |

Models of nucleotide evolution: K80 – Kimura (1980), GTR – *General Time Reversible* (Tavaré, 1986), e HKY - Hasegawa-Kishino-Yano (Hasegawa et al. 1985). I – proportion of invariable sites proportion, G – Gamma distribution (Yang, 1996).
