# Supplementary material for: Cryptic Genetic Diversity Is Paramount in Small-Bodied Amphibians of the Genus Euparkerella (Anura: Craugastoridae) Endemic to the Brazilian Atlantic Forest
Source: PLoS One. 2013 Nov 1;8(11):e79504. doi: 10.1371/journal.pone.0079504 (PMC3815154; doi:10.1371/journal.pone.0079504)
Supplement: Protocol S1 — (DOCX) [file pone.0079504.s002.docx]

**Protocol S1**

*DNA extraction, amplification and sequencing*

Tissues were digested in lysis buffer solution with Proteinase K and purified with DNeasy mini spin columns using DNeasy Blood & Tissue kit (QIAGEN, Hilden, Germany), following the manufacturers protocol.

Pairs of primers used in PCR reactions are listed in Table S2. Amplification reactions for CO-I were performed with 1µl of DNA extraction product, 10x Taq buffer, 0.2 µM of each primer, 1.45 mM of MgCl_2_, 200 µM of each dNTPs, and 0.5 U of Taq DNA polymerase (recombinant) (Fermentas International Inc., Burlington, ON, Canada) in a 13 µl final volume product. PCR cycling conditions were as follows: 3 minutes of initial denaturation at 94º C, 35 cycles of 94º C for 20 s, 50º C for 20 s and 60º C for 1min and 30 s, with a final extension at 60º C for 5 min. For nuclear genes we used nested PCR. The first amplification of C-myc2 were performed with 1µl of DNA extraction product, 10x Taq buffer, 0.45 µM of each primer, 2.75 mM of MgCl_2_, 115 µM of each dNTPs, and 0.1 U of Taq DNA polymerase in a 11 µl final volume product. In the second reaction we used 1 µl of amplification product of first reaction and changed the MgCl_2_ concentration to 1.7 mM. PCR cycling conditions in the first reaction were: 3 minutes of initial denaturation at 94º C, 30 cycles of 94º C for 20 s, 48º C for 20 s and 72º C for 1min, with a final extension at 72º C for 5 min. In the second reaction we increased the number of cycles to 35 and annealing temperature to 52º C. For other nuclear genes, β-fibint7, RAG-1 e TYR, PCRs conditions were the same. The first amplification were performed with 1µl of DNA extraction product, 10x Taq buffer, 0.25 µM of each primer, 1.7 mM of MgCl_2_, 230 µM of each dNTPs, and 0.06 U of Taq DNA polymerase in a 11 µl final volume product. In the second reaction we used 1 µl of amplification product of first reaction and kept the same reagents concentration. PCR cycling conditions in the first reaction were: 3 minutes of initial denaturation at 94º C, 20 cycles of 94º C for 20 s, 50º C for 20 s and 72º C for 1min and 30s, with a final extension at 72º C for 5 min. For the second reaction we increased the annealing temperature to 52º and the number of cycles to 35.

PCR products were purified in a 0,3 µl ExoI, 0,6 µl FasAP solution (Fermentas International Inc., Burlington, ON, Canada) and 1,1 µl of ultrapure water, reacting at 37º C for 30 min and 95º C for 10 min. Sequencing reactions were performed as follow: 2 µl of Save Money buffer (200 mM Tris–HCl, pH 9.0; 5 mM MgCl), 0,4 µl of Big Dye v.3.1. (Applied Biosystems, USA), 0,32 µl of primers (10pmol/ µl), 5-20ng of PCR product, and 10 µl of water ultrapure. PCR thermal conditions were: 95º C for 1 min, followed by 29 cycles of 95º C for 20 s, 50º C for 20 s, and 60ºC for 4 min. Samples were read in an ABI3500 sequencer (Applied Biosystems, USA).
